# Direct interfacial Y_731_ oxidation in α_2_ by a photoβ_2_ subunit of *E. coli* class Ia ribonucleotide reductase†
†Electronic supplementary information (ESI) available: Experimental procedures, calculation of uncertainty in data analysis, purity gels, determination of *K*_D_, spectroscopic characterization, time-resolved emission decay traces, and table from reference S8 are provided. See DOI: 10.1039/c5sc01125f



**DOI:** 10.1039/c5sc01125f

**Published:** 2015-06-08

**Authors:** David Y. Song, Arturo A. Pizano, Patrick G. Holder, JoAnne Stubbe, Daniel G. Nocera

**Affiliations:** a Department of Chemistry and Chemical Biology , 12 Oxford Street , Cambridge , MA 02138-2902 , USA . Email: dnocera@fas.harvard.edu; b Department of Chemistry , Massachusetts Institute of Technology , 77 Massachusetts Avenue , Cambridge , MA 02139-4307 , USA . Email: stubbe@mit.edu

## Abstract

Proton-coupled electron transfer (PCET) is a fundamental mechanism important in a wide range of biological processes including the universal reaction catalysed by ribonucleotide reductases (RNRs) in making *de novo*, the building blocks required for DNA replication and repair. These enzymes catalyse the conversion of nucleoside diphosphates (NDPs) to deoxynucleoside diphosphates (dNDPs). In the class Ia RNRs, NDP reduction involves a tyrosyl radical mediated oxidation occurring over 35 Å across the interface of the two required subunits (β_2_ and α_2_) involving multiple PCET steps and the conserved tyrosine triad [Y_356_(β_2_)–Y_731_(α_2_)–Y_730_(α_2_)]. We report the synthesis of an active photochemical RNR (photoRNR) complex in which a Re(i)-tricarbonyl phenanthroline ([Re]) photooxidant is attached site-specifically to the Cys in the Y_356_C-(β_2_) subunit and an ionizable, 2,3,5-trifluorotyrosine (2,3,5-F_3_Y) is incorporated in place of Y_731_ in α_2_. This intersubunit PCET pathway is investigated by ns laser spectroscopy on [Re_356_]-β_2_:2,3,5-F_3_Y_731_-α_2_ in the presence of substrate, CDP, and effector, ATP. This experiment has allowed analysis of the photoinjection of a radical into α_2_ from β_2_ in the absence of the interfacial Y_356_ residue. The system is competent for light-dependent substrate turnover. Time-resolved emission experiments reveal an intimate dependence of the rate of radical injection on the protonation state at position Y_731_(α_2_), which in turn highlights the importance of a well-coordinated proton exit channel involving the key residues, Y_356_ and Y_731_, at the subunit interface.

## Abstract 2

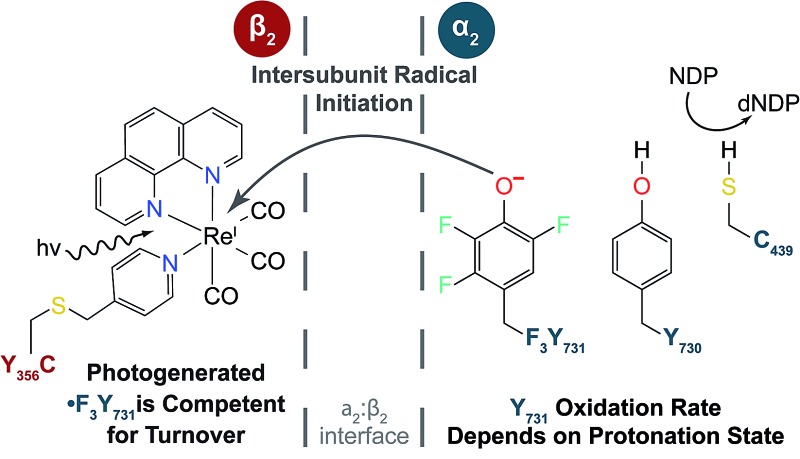
Proton-coupled electron transfer (PCET) is a fundamental mechanism important in a wide range of biological processes including the universal reaction catalysed by ribonucleotide reductases (RNRs) in making *de novo*, the building blocks required for DNA replication and repair.

## Introduction

Enzymatic control of coupled proton and electron transfer^1–4^ is critical in managing biological processes ranging from energy storage (photosystem II)^5–9^ and conversion (cytochrome c oxidase)^10^ to the synthesis of DNA precursors (ribonucleotide reductase).^11–14^ To better understand biological PCET, we have undertaken studies of this process in class Ia RNRs, which catalyse the conversion of nucleoside diphosphates (NDPs) to deoxynucleoside diphosphates (dNDPs)—a process required for synthesis and repair of DNA in all organisms.^15,16^ Catalysis by the class I RNRs proceeds by a radical mechanism requiring coupling of radical transport over 35 Å involving PCET across the two subunits to substrate turnover. The long distance, reversibility, and rate-limiting conformational gating of radical transport have made study of this process challenging. To overcome this challenge, we have developed two methodologies: photoRNRs^17–21^ and site-specific incorporation of unnatural amino acids in place of pathway residues.^22–24^

**Fig. 1:**
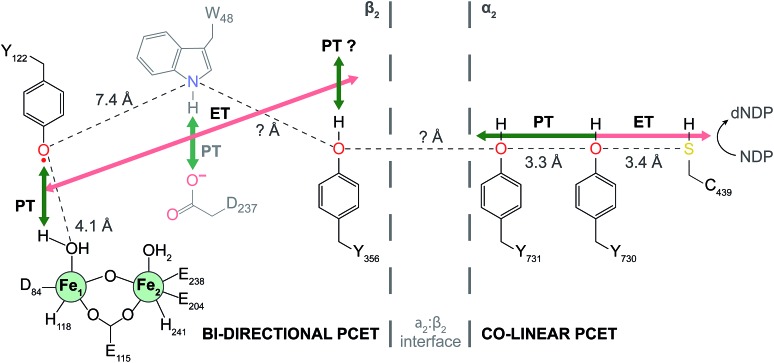
Current model of radical transport pathway in class Ia RNR leading to nucleotide reduction as determined by the docking model^16^ and diagonal distance measurements acquired by PELDOR spectroscopy.^34^ Key redox active amino acids and known distance measurements involved in PCET pathway are shown. Residue W_48_ is grayed indicating the absence of experimental evidence supporting its participation in the PCET radical mechanism. Residue Y_356_ is shown at the interface for illustrative purposes. Salmon arrows indicate electron transfer (ET) and green arrows represent proton transfer (PT). In α_2_, co-linear PCET is denoted by the dual-colored arrow, and the proposed bi-directional PCET in β_2_ is indicated by the orthogonality of the ET and PT pathways.

*E. coli* class Ia RNR has served as the paradigm for this long distance radical transport. It is composed of two homodimeric subunits: α_2_ and β_2_. A docking model for this complex,^25^ substantiated by recent biochemical and biophysical studies,^26,27^ has provided the working model for the radical transport pathway shown in Fig. 1. The active site for NDP reduction resides in α_2_, where the cysteine radical (C_439_˙) must be transiently generated during each turnover by the essential diferric-tyrosyl radical (Y_122_˙) cofactor in β_2_. This long range oxidation requires a multi-step radical hopping mechanism that involves a specific pathway including four tyrosines (Y_122_ and Y_356_ in β_2_; Y_731_ and Y_730_ in α_2_)^11,28^ and potentially W_48_ in β_2_.^11^

Recent attention has focused on the detection of the proposed transient radical intermediates and identification of the operative PCET mechanism at each site. Mössbauer studies have established that Y_122_˙ reduction in β_2_ is triggered by binding of substrate and effector to α_2_ ^29^ and involves proton donation from the water at Fe_1_ (Fig. 1). This process involves orthogonal PCET wherein the proton and electron come from different residues. High-field electron paramagnetic resonance (Hf EPR) and deuterium electron nuclear double resonance (ENDOR) have provided atomic level resolution of local hydrogen bond interactions, specifically the co-linearity of the PCET within α_2_. Additionally, significant shifts in *g*_x_ values together with the assignment of hyperfine coupling features from the ENDOR spectra of various amino-substituted RNR mutants propose an important role for electrostatics at the α_2_:β_2_ interface.^30^ However, the disordered C-terminal tail of β_2_ where Y_356_ resides has made interrogation of the chemistry at the subunit interface challenging (Fig. 1).

Rate limiting conformational gating in RNR obscures radical transport across the subunit interface, prompting us to develop photoRNRs to trigger radical initiation with light to avoid this gating and to potentially enable the observation of Y˙ at the interface. Radical injection kinetics were initially made possible using a 19mer peptide photoRNR, which corresponded to the identical 19 residues of the C-terminal tail of β_2_ along with a modification that appended a photooxidant (rhenium phenanthroline [Re]) adjacent to Y_356_ or fluorinated derivatives. This peptide photoRNR enables nucleotide reduction in the presence of α_2_ and light and allows for observation of radical injection into α_2_.^21^ Radical injection was only realized in the presence of an intact Y_731_–Y_730_ dyad within α_2_, providing important support for co-linear PCET within this subunit.

More recently, photoRNRs have been generated in which the peptide with the photooxidant is replaced by the full-length β_2_ containing a site-specifically incorporated [Re] photooxidant at residue 355 using a S_355_C-β_2_ mutant.^31^ Transient absorption spectroscopy on the active [Re_355_]-β_2_:α_2_ complex in comparison to the control [Re_355_]-Y_356_F-β_2_:α_2_ complex allowed for the assignment of a photogenerated ˙Y_356_.^32^ In addition, further modification of [Re_355_]-β_2_ by installation of an unnatural 2,3,5-F_3_Y in place of Y_356_ in β_2_ yielded the first direct measurement of ˙Y propagation kinetics through the active RNR complex.^33^ Comparison of ˙Y decay transient kinetics in the [Re_355_]-2,3,5-F_3_Y_356_-β_2_:α_2_ complex in the presence of substrate, either [3′-^1^H]-CDP or [3′-^2^H]-CDP, and effector ATP, revealed an observed rate constant of 1.4 × 10^4^ s^–1^ and unmasked for the first time an isotope effect on cleavage of the 3′ C–H of the substrate. The photoRNRs thus circumvent the masking of radicals by conformational gating and thus have provided insight regarding radical transport and nucleotide reduction chemistry not accessible by any other method.

In this work, the [Re] photooxidant is attached to Cys in the Y_356_C-β_2_ mutant and this modified [Re_356_]-β_2_ subunit is associated to α_2_ in which Y_731_ is site-specifically replaced with 2,3,5-F_3_Y. This [Re_356_]-β_2_:2,3,5-F_3_Y_731_-α_2_ complex together with the [Re_356_]-β_2_:wt-α_2_ and [Re_356_]-β_2_:Y_731_F-α_2_ control complexes are studied in the presence of CDP and ATP. In contrast to previous photoRNR systems, installation of [Re] at position 356 enables direct interfacial generation of a tyrosyl radical at position 731. Additionally, by leveraging the greater acidity of 2,3,5-F_3_Y to enable deprotonation at neutral pH, this residue furnishes an ionizable reporter that varies with experimental pH. In turn, site-selective removal of a single proton at position Y_731_(α) provides the first protein:protein scaffold of RNR that permits the investigation of the effect of a modified proton microenvironment on radical transport on transient time scales (sub μs) at the interface. In the absence of Y_356_, radical injection is only achieved when position Y_731_ is deprotonated. In addition to confirming the complexity of RNR in maintaining a well-organized PCET pathway, this work introduces and highlights the importance of a well-defined proton exit channel out of α_2_ involving the key pathway residues, Y_356_ and Y_731_, at the subunit interface.

## Experimental

Modified RNR subunits were constructed, expressed, purified, modified, and characterized as previously reported or with minor modification.^19,23,24,31,35^ Protein concentrations were measured by absorbance at 280 nm using: *ε*_α_2__ = 189 000 M^–1^ cm^–1^, *ε*_β_2_-apo_ = 121 000 M^–1^ cm^–1^, *ε*_β_2_-holo_ = 131 000 M^–1^ cm^–1^, and *ε*_β_2_-[Re]_ = 189 000 M^–1^ cm^–1^. Purity of protein constructs was assessed by SDS-PAGE (Fig. S1†). All measurements were conducted in assay buffer at pH 7.6 (50 mM HEPES, 15 MgSO_4_, 1 mM EDTA; unless otherwise specified). Measurement of the dissociation constant (*K*_D_) between [Re_356_]-β_2_ and wt-α_2_ was performed by a spectrophotometric competitive inhibition assay as previously reported.^17,21^ Measurement of the p*K*_a_ of the phenolic proton of 2,3,5-F_3_Y_731_ within the assembled 2,3,5-F_3_Y_731_-α_2_:[Re_356_]-β_2_ complex was performed by fluorometric titration as previously reported.^21^ The details of methods that deviate from published procedures are provided in the ESI.† Similarly, photoinitiated nucleotide reduction activity assays were performed according to published methods.^17–19,32^ Error bars represent 2*σ* resulting from photolysis on ≥three independent samples.

Time-resolved spectroscopic measurements were performed using a home-built nanosecond laser system previously described.^21,31–33^ Each sample was prepared prior to photolysis and measurements were performed in triplicate. The calculation of the uncertainty in experimental measurements to 95% confidence limits (2*σ*) is described in the ESI (eqn S1–S5†).

## Results and discussion

### PhotoRNRs: [Re_356_]-β_2_:α_2_ and [Re_356_]-β_2_:2,3,5-F_3_Y_731_-α_2_

**Fig. 2:**
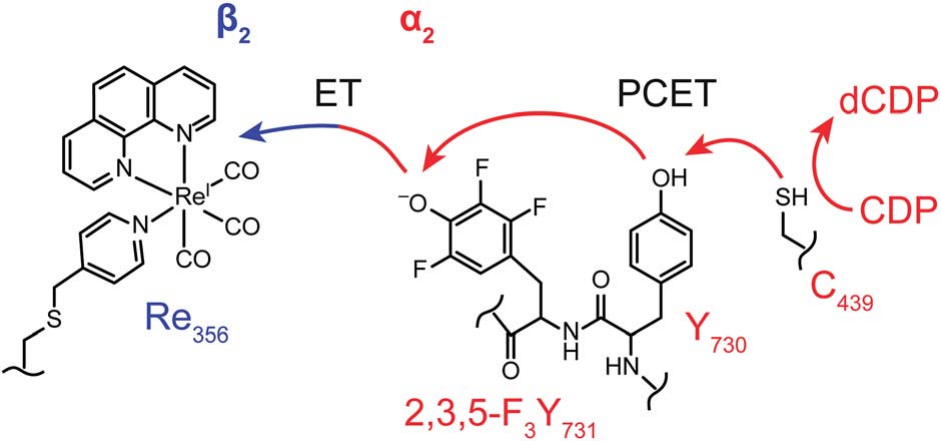
PhotoRNR design schematic depicting the intersubunit photochemical oxidation of 2,3,5-F_3_Y_731_ by [Re_356_]-β_2_, incorporation of 2,3,5-F_3_Y at position Y_731_ allowed radical generation to be examined in the absence of a proton at this residue.

To probe radical initiation across the α_2_:β_2_ interface, specific variants of each subunit were required. To directly target the intersubunit radical transport step of Y_731_ oxidation and subsequent radical injection into α_2_, we chose to circumvent Y_356_ oxidation entirely. In contrast to previous systems where photooxidants were placed adjacent to Y_356_ at position 355, [Re] replaces Y_356_ in this study. The new construct maintains the mutations C_268_S and C_305_S, and preserves catalytic activity. Additionally, the mutation, Y_356_C, thus enables alkylation with [Re]-Br to yield [Re_356_]-β_2_. To examine the effect of a proton at position Y_731_, this residue was replaced with 2,3,5-F_3_Y, solution p*K*_a_ = 6.4, compared with Y (p*K*_a_ = ∼10). The photoRNR construct is illustrated in Fig. 2. These two subunit modifications allow for direct oxidization of Y_731_ by a photoβ_2_.

Construction, expression, isolation, and labelling to generate [Re_356_]-β_2_ were performed as previously reported with minor modifications.^31^ Unlabelled and reconstituted Y_356_C-β_2_ (holo) is inactive (0.16(5) U) towards nucleotide reduction (wt-β_2_ activity = 6000–8000 U), as expected given the absence of Y_356_. We note that labelling does not preclude binding (*K*_D_, = 0.43(11) μM), as measured in the competitive inhibition assay shown in Fig. S2.† This value is in agreement with previously reported values for active photoRNR β_2_ mutants,^32^ and not significantly altered from wt-RNR.^12,36^

[Re_356_]-β_2_ displays similar spectroscopic and photophysical properties, as reported previously for [Re_355_]-β_2_.^31^ Fig. S3† shows typical absorption and emission spectra for [Re_356_]-β_2_; the absorption spectrum is dominated by the characteristic features of rhenium(i) tricarbonyl bisimine compounds and the emission originates from a triplet metal-to-ligand charge transfer state. The absorption spectrum of [Re_356_]-β_2_ is accurately re-constructed as the sum of Y_356_C-β_2_ and twice the [Re]-Br absorption spectrum, as expected for a construct with two [Re] molecules per β_2_ dimer (Fig. S3†).

Construction of 2,3,5-F_3_Y_731_-α_2_ was achieved using *in vivo* nonsense suppression methodology previously developed to install fluorotyrosine reporters in class Ia RNR.^24^ The specific activity of 2,3,5-F_3_Y_731_-α_2_ under catalytic conditions (375(5) U) is diminished relative to wt-α_2_ (1800–2500 U), while under single-turnover activity (2.75(4) equiv. dCDP/α_2_) is comparable to wt-α_2_ (∼3 dCDP equiv./α_2_). This decrease in catalytic activity, though comparable to that of wild-type (wt), is consistent with previous reports of this mutant.^37^

**Fig. 3:**
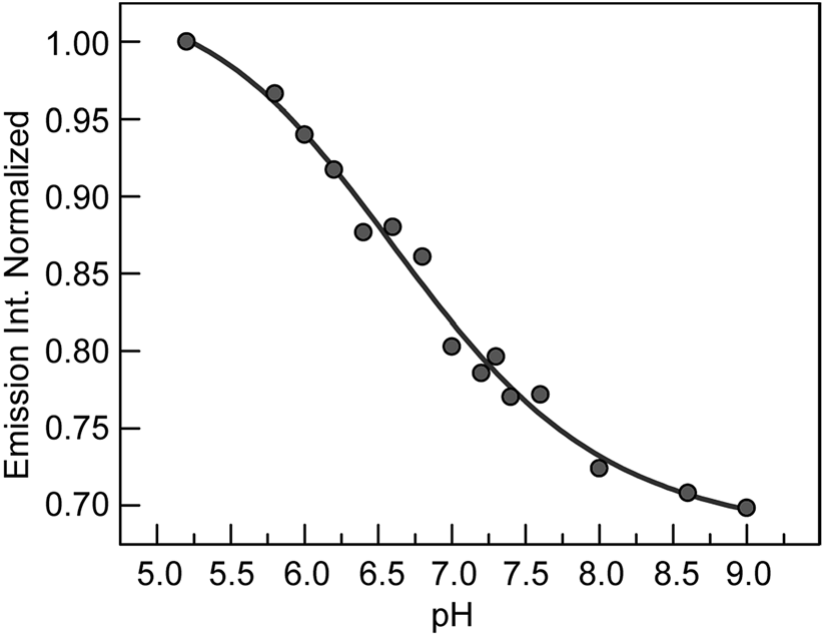
Measurement of the p*K*_a_ of the phenolic proton of 2,3,5-F_3_Y_731_ in the [Re_356_]-β_2_:2,3,5-F_3_Y_731_-α_2_ complex by steady-state emission. Fluorometric titrations of 2.5 μM 2,3,5-F_3_Y_731_-α_2_, 3.75 μM [Re_356_]-β_2_, 1 mM CDP, and 3 mM ATP were carried over a pH range of 5 to 9. Samples were illuminated with *λ*_exc_ = 315 nm, scanned from 420–700 nm at 0.5 nm intervals, integrated for 1 s at each data point, and averaged from three scans. The collected emission plots were integrated for fluorescence intensity and plotted against pH. Data were fit to an internal sigmoidal logistic function in OriginPro 8.5. The inflection point of the monoprotic pH-titration curve (*x*_0_) is the p*K*_a_:
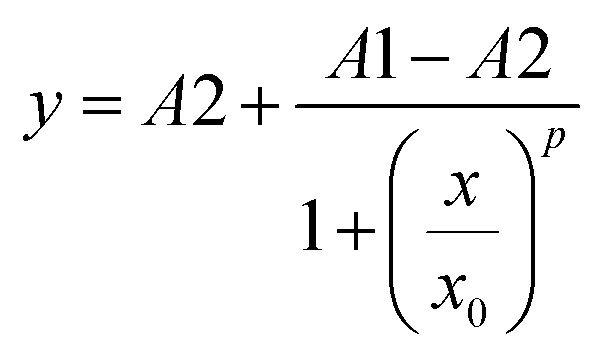
.

The deprotonation of 2,3,5-F_3_Y_731_ is expected to perturb the rate of radical generation at position Y_731_. The kinetic penalty associated with proton transfer is alleviated by removal of the proton when experimental pH > p*K*_a_, thus requiring measurement of the precise p*K*_a_ of 2,3,5-F_3_Y_731_ in the assembled construct. As previously reported,^19–21,32,33,38^ the increase in the rate of photooxidation for tyrosinate relative to tyrosine,^39^ makes [Re] emission a reporter of the protonation state of nearby tyrosine residues. Fluorometric titration of the [Re_356_]-β_2_:2,3,5-F_3_Y_731_-α_2_ complex reveals the p*K*_a_ of 2,3,5-F_3_Y_731_ to be 6.7(1), Fig. 3. Accordingly, ∼90% of 2,3,5-F_3_Y_731_ residues are deprotonated under experimental conditions at the optimal operating pH for RNR (pH 7.6). Given the thermodynamically unfavourable acidity of the tyrosyl radical cation (p*K*_a_ = –2) a PCET process managing both the electron and proton transfers is mandated.^40,41^

### Photoinitiated substrate turnover

**Fig. 4:**
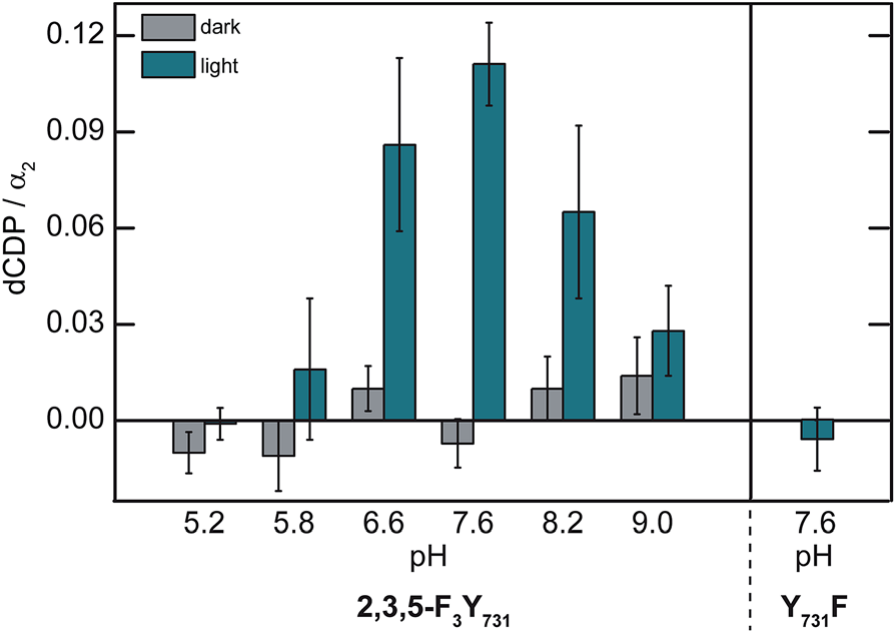
Photoinitiated nucleotide reduction assay monitored (left) as a function of pH in the [Re_356_]-β_2_:2,3,5-F_3_Y_731_-α_2_ complex and (right) at pH = 7.6 for the control, [Re_356_]-β_2_:Y_731_F-α_2_. A solution containing 25 μM [Re_356_]-β_2_, 200 μM [^3^H]-CDP (21 148 cpm nmol^–1^), 3 mM ATP in assay buffer, and a separate solution of 10 μM α_2_ were incubated at 25 °C. Following two min incubation the two solutions were mixed, transferred to the photolysis cuvette, and illuminated (*λ* > 313 nm) for 10 min.

To establish that the photoRNR construct is competent to generate dCDP, the [Re_356_]-β_2_:2,3,5-F_3_Y_731_-α_2_ complex in the presence of [^3^H]-CDP and ATP was photolyzed for 10 min (*λ* > 313 nm) and dCDP was measured by scintillation counting. The results of this single turnover experiment are shown in Fig. 4. Perturbation of the enzyme by the introduction of [Re] results in a reduced level of turnover that is 5–10% relative to wt-RNR under the same pH conditions. Notwithstanding, the presence of photogenerated products establish the relevance of [Re_356_]-β_2_:2,3,5-F_3_Y_731_-α_2_ complex to the natural enzyme. Attenuated enzymatic activity is also detected at pH > 7.6 which is consistent with the observed pH rate profiles for the wt enzyme and fluorotyrosine derivatized RNR constructs.^42^

### Radical injection kinetics

The photogeneration of product for the [Re_356_]-β_2_:2,3,5-F_3_Y_731_-α_2_ complex prompted us to undertake radical injection kinetics studies. Using the [Re]* emission lifetime as a reporter for radical injection, ns TA laser spectroscopy on the [Re_356_]-β_2_:α_2_ complexes in the presence of CDP and ATP was conducted. The emission decay lifetimes for each construct were measured at pH = 7.6, where maximum turnover was observed, and are summarized in Table 1; representative traces are included in Fig. S4.† As previously observed for [Re_355_]-β_2_,^32^ the [Re]* lifetime in [Re_356_]-β_2_ (*τ* = 507(3) ns) increases upon binding to Y_731_F-α_2_ (*τ*_o_ = 652(4) ns), consistent with the more hydrophobic environment engendered by the protein environment. The lifetime *τ*_o_ of the Y_731_F-α_2_ variant provides a reference for excited-state decay of [Re]* when it is located at the interface, but in the absence of quenching by the tyrosine located at position 731. Upon introduction of Y_731_, the [Re]* emission (*τ* = 629(4) ns) is quenched relative to Y_731_F-α_2_ according to the following equation:1
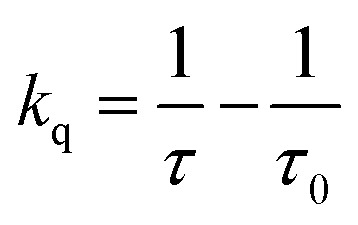



**Table 1:** Radical injection rates from [Re_356_]-β_2_ to α_2_ variants modified at position 731 at pH = 7.6

[Re_356_]-β_2_: **X**_731_-α_2_	*τ*_600 nm ns^–1^_ ^*a*^	*k*_q_/10^4^ s^–1^ ^*b*^
**F**	652(4)	—
**Y**	629(4)	5.6 (1.4)
**2,3,5-F**_3_ **Y**	593(6)	15.4 (2.2)

^*a*^Lifetime of emission decay measured on 10 μM [Re_356_]-β_2_, 25 μM α_2_ (as indicated), 1 mM CDP, 3 mM ATP in assay buffer (pH 7.6), *λ*_exc_ = 355 nm, *λ*_obs_ = 600 nm. Errors shown in parentheses represent 2*σ* resulting from measurement on ≥3 independent samples.

^*b*^Emission quenching rate constant, *k*_q_, determined from eqn (1). Error in quenching rate constants calculated as shown in ESI.

Accordingly, this quenching rate constant, *k*_q_, is equivalent to the radical generation rate, and from eqn (1) it is calculated to be 5.6(1.4) × 10^4^ s^–1^. In comparison to previously observed photooxidation of Y_356_ in [Re_355_]-β_2_ (*k*_q_ = 4.1(1) × 10^5^ s^–1^), the direct oxidation of Y_731_ by [Re_356_]*-β_2_ is slower possibly owing to an increased charge transfer distance, which is occurring across the subunit interface *vs.* at adjacent positions in previous investigations. Further analysis of the emission decay kinetics of [Re]* reveal a dependency on the rate of radical injection and the protonation state of tyrosine at position Y_731_(α). Replacement of Y_731_ with 2,3,5-F_3_Y_731_ enhances the quenching rate constant by a factor of three (*τ* = 593(7) ns (*k*_q_ = 15.4(2.2) × 10^4^ s^–1^). This acceleration of radical injection into α_2_ is in accordance with the differing protonation states of tyrosine in the two constructs. Under the experimental conditions of pH = 7.6, 2,3,5-F_3_Y_731_ is deprotonated and hence quenching occurs by ET rather than PCET, which results in faster tyrosine oxidation, despite being ∼50–100 mV more difficult to oxidize than native tyrosine at this pH (as determined from solution peak potentials (*E*_p_) of N-acetyl and C-amide protected fluorotyrosines measured by differential pulse voltammetry).^22^ For clarity, obtaining precise single reside midpoint potentials in protein constructs is extremely challenging, and thus thermodynamic considerations must be guided from these measured *E*_p_ values,^11^ despite recent findings that suggest significant deviations of the formal reduction potential and solution *E*_p_ values.^43^

**Fig. 5:**
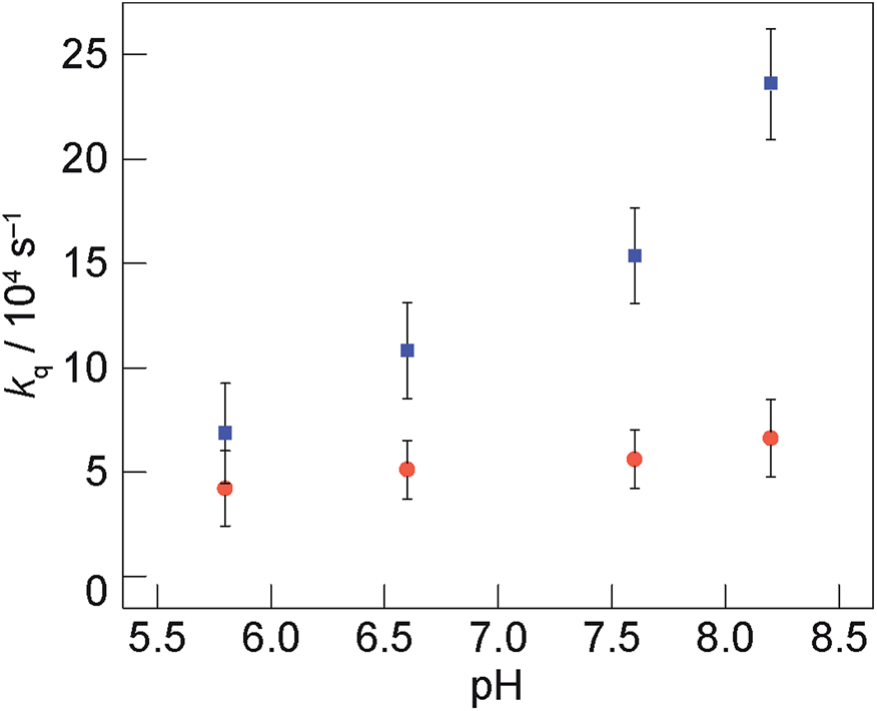
Quenching rate constant of [Re]* by tyrosine, *k*_q_, monitored as function of pH: Y_731_(wt) relative to Y_731_F (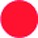) and 2,3,5-F_3_Y_731_ relative to Y_731_F (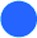). *τ*_o_ corresponds to the lifetime of [Re_356_]-β_2_:Y_731_F-α_2_. Error bars in *k*_q_ represent 2*σ* resulting from error propagation on lifetime measurements as described in ESI.†

To investigate how the proton at position Y_731_ affects interfacial PCET and subsequent interfacial radical injection kinetics, emission quenching of the [Re]* within the [Re_356_]-β_2_:2,3,5-F_3_Y_731_-α_2_ complex was monitored over the activity accessible pH region of RNR. A plot of the [Re]* decay lifetimes for [Re_356_]-β_2_ alone and in the three [Re_356_]-β_2_:α_2_ variant complexes monitored as a function of pH are shown in Fig. S5;† representative emission decay traces are provided in Fig. S4.† The quenching rate constants, *k*_q_, as a function of pH may be determined from eqn (1); these rate constants are plotted in Fig. 5. Quenching by Y_731_(wt)-α_2_ (red circles, 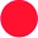) and 2,3,5-F_3_Y_731_-α_2_ (light blue squares, 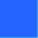) are referenced to the control, Y_731_F-α_2_. Within our error limits, little to no dependence of *k*_q_ is observed for the [Re_356_]-β_2_:Y_731_(wt)-α_2_ complex (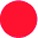), as native tyrosine is protonated throughout the pH window. Conversely, a large pH dependence is observed for *k*_q_ when [Re_356_]-β_2_:2,3,5-F_3_Y_731_-α_2_ is compared to Y_731_F (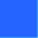). Guided by our measurement of the p*K*_a_ of 2,3,5-F_3_Y_731_-α_2_, shown in Fig. 3, we ascribe the observed differences in quenching to the relative ratio of deprotonated:protonated forms of 2,3,5-F_3_Y_731_ as pH is varied. The large *k*_q_s at high pH is expected owing to deprotonation of F_3_Y_731_ whereas quenching at low pH approaches that of Y_731_ where both of the tyrosines are protonated.

**Scheme 1:**
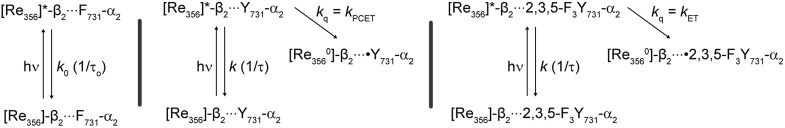
Excited-state deactivation pathways for [Re_356_]-β_2_ in the presence of indicated α_2_ subunit.

Scheme 1 summarizes the decay pathways for [Re]* in the different variants. The presence of tyrosine introduces an excited-state decay pathway *via* radical generation. For the case of Y_731_(wt)-α_2_, oxidation occurs by PCET whereas for 2,3,5-F_3_Y_731_-α_2_ occurs by ET. These differing mechanisms of tyrosine oxidation for the two variants in the absence of Y_356_ introduce the possibility that Y_356_ may be involved with facilitating proton removal at the interface, though future experiments are needed to establish its specific role. While a co-linear Y_356_–Y_731_ π-stacked mode where Y_356_ acts directly as the proton acceptor for Y_731_ is unlikely in light of recent Hf EPR/ENDOR data on amino-substituted tyrosine derivatives at various pathway positions, the strongly perturbed *g*_x_ values of NH_2_Y_356_˙ indicate that Y_356_ may communicate with Y_731_ through a network of water molecules.^30^ Additional evidence supporting the involvement of Y_356_ in modulating Y_731_ oxidation was also observed in [Re_355_]-β_2_ construct.^32^ The photooxidation kinetics of Y_731_, which are summarized in Table S1,† indicate that Y_731_ radical generation is enhanced by the presence of Y_356_ as [Re_355_]-β_2_ oxidation of Y_731_ is 2.1(1.2) times faster for Y_356_ than for F_356_. The presence of Y_356_ may facilitate proton removal from α_2_ *via* the interface, thus assisting in PCET.

In this directed study, whereby Y_356_ is absent by virtue of its replacement with [Re_356_], efficient injection of a radical into α_2_ is realized only when a proton is removed from the pathway by the introduction of 2,3,5-F_3_Y_731_. While this result does not implicate Y_356_ directly as the proton acceptor for Y_731_, it supports the contention that Y_356_ is in communication with Y_731_ at the α_2_:β_2_ subunit interface and that Y_356_ enables the PCET required for efficient radical transport. Further investigations of this contention are underway along with studies to assess the role of possible contributions from other residues, or perhaps metal ions that may also be involved in managing protons at the interface.

## Conclusions

Replacement of Y_356_ by a [Re] photooxidant and installation of 2,3,5-F_3_Y at position 731 in α_2_ furnishes a photoRNR that specifically targets intersubunit radical transport. This construct supports photoinitiated substrate turnover, confirming its fidelity to the natural system. Time-resolved emission studies reveal that 2,3,5-F_3_Y_731_ is oxidized at a rate 3 times faster than native Y_731_ even though the non-natural amino acid is thermodynamically more difficult to oxidize at pH 7.6 (Δ*E*_p_ ∼ 50–100 mV). These results emphasize the enzymatic imperative for coupling the proton and electron to allow for efficient radical transport. In conjunction with the parallel studies of [Re_355_]-β_2_, these results suggest the importance of a well-coordinated proton exit channel involving Y_356_ and Y_731_ as key interfacial residues for radical transport across the α_2_:β_2_ interface.
