# Supplementary material for: Direct interfacial Y731 oxidation in α2 by a photoβ2 subunit of E. coli class Ia ribonucleotide reductase
Source: Chem Sci. 2015 Jun 8;6(8):4519–24. doi: 10.1039/c5sc01125f (PMC4618407; doi:10.1039/c5sc01125f)
Supplement: Supplementary file 1 [file SC-006-C5SC01125F-s001.pdf]

Electronic Supplementary Material (ESI) for Chemical Science.
This journal is © The Royal Society of Chemistry 2015

Supplementary Information

Direct Interfacial Y_{731} Oxidation in α_2 by a Photoβ_2 Subunit of *E. coli* Class Ia Ribonucleotide Reductase

David Y. Song,[a] Arturo A. Pizano,[a] Patrick G. Holder,[a] JoAnne Stubbe,[b]* and Daniel G. Nocera[a]*

[a] *Department of Chemistry and Chemical Biology, Harvard University, 12 Oxford Street, Cambridge, MA 02138;* [b] *Department of Chemistry, Massachusetts Institute of Technology, 77 Massachusetts Avenue, Cambridge, MA 02139*

stubbe@mit.edu, dnocera@fas.harvard.edu

Index *Page*

Experimental methods S2

Materials, molecular biology, synthesis and buffers S2

Biochemical details Biophysical measurements S3

Biophysical measurements S4

Data analysis and error propagation S5

Figure S1. Expression and purity gels of RNR subunits S6

Figure S2. Measurement of K_D S7

Figure S3. Absorption and emission spectra of constructs S8

Figure S4. Transient decay traces S9

Figure S5. Plot of lifetimes vs. pH S10

Table S1. Kinetics data for $[Re_{355}]$-Y_{356} construct S11

Experimental methods

Materials. XL 10-Gold Ultracompetent cells (Agilent), kanamycin (Km) (Sigma-Aldrich), Spin Miniprep Kit (QIAgen), BL21(DE3) competent cells (EMD Millipore), 1,10-phenanthroline (phen) (Strem), glycerol (BDH), Luria-Bertani media (LB) (BD Biosciences), acetone (Sigma-Aldrich), acetonitrile (MeCN) (Sigma-Aldrich), isopropyl *β*-D-1-thiogalactopyranoside (IPTG) (Sigma-Aldrich), phenylmethylsulfonly fluoride (PMSF) (Sigma-Aldrich), dithiothreitol (DTT) (Sigma-Aldrich), hydrochloric acid (HCl) (Sigma-Aldrich), tris(hydroxymethyl)aminomethane (Tris) (EMD Millipore), streptomycin sulfate (strep) (Sigma-Aldrich), Ni^{2+}-nitrilotriacetic acid (Ni-NTA) superflow resin (QIAgen), Imidazole (Sigma-Aldrich), sodium chloride (NaCl) (EMD), Amicon Ultrafitration Discs YM-30 (EMD Millipore), 4-(2-hydroxyethyl)-1-piperazineethane sulfonic acid (HEPES) (molecular biology grade, EMD Millipore), 2-(N-morpholino)ethanesulfonic acid (MES) (EMD Millipore), 3-[[1,3-dihydroxy-2-(hydroxymethyl)propan-2-yl]amino]propane-1-sulfonic acid (TAPS), Bradford reagent (Bio-RAD), Amicon Ultra-15 centrifugal filter unit with ultracel-30 membrane (EMD Millipore), ethanol (EtOH) (Sigma-Aldrich), hydroxyurea (HU) (Sigma-Aldrich), N,N-dimethylformamide (DMF) (Sigma-Aldrich), ethylenediamine-tetraacetic acid (EDTA) (Mallinckrodt), 2,3,6-trifluorophenol (Sigma-Aldrich), pyridoxal-5′-phosphate (PLP) (Sigma-Aldrich), 5,5′-dithiobis(2-nitrobenzoic acid) (DTNB) (Sigma-Aldrich), ddH_20 (18 MΩ), 7.5% Mini-PROTEAN TGX Precast Gel (Bio-Rad), Precision Plus Protein Standard (Bio-Rad).

Molecular biology and synthesis. Plasmids encoding for the fluorotyrosine tRNA synthetase (pEVOL-2,3,5-F_3Y-RS),[1] the α_2 subunit with TAG codon at position 731 (pET28a-nrdA-Y_{731}Z-TAG),[1] tyrosine phenol lyase (TPL),[2] and the β_2 subunit lacking surface accessible cysteine residues (pET9d-His_6-C_{268}S-C_{305}S-nrdB)[3] were available from previous studies. Primers for site-directed mutagenesis were purchased from Invitrogen. Glycerol stocks for wt-β_2, wt-α_2, and Y_{731}F-α_2, were available from a previous study. 4-Bromomethylpyridyltricarbonyl(1,10)-phenanthrolinerhenium(I) [Re]-Br[3] and 2,3,5-trifluorotyrosine (2,3,5-F_3Y)[4] were synthesized as previously described.

---

1 Incorporation of fluorotyrosines into ribonucleotide reductase using an evolved, polyspecific aminoacyl-tRNA synthetase. E.C. Minnihan, D.D. Young, P.G. Schultz, J. Stubbe, *J. Am. Chem. Soc.* 2011, **133**, 15942–15945.

2 Direct observation of a transient tyrosine radical competent for initiating turnover in a photochemical ribonucleotide reductase. S.Y. Reece, M.R. Seyedsayamdost, J. Stubbe, D.G. Nocera, *J. Am. Chem. Soc.* 2007, **129**, 13828–13830.

3 Photo-ribonucleotide reductase β_2 By selective cysteine labeling with a radical phototrigger. A.A. Pizano, D.A. Lutterman, P.G. Holder, T.S. Teets, J. Stubbe, D.G. Nocera, *Proc. Natl. Acad. Sci. U.S.A.* 2012, **109**, 39–43.

4 Site-specific incorporation of fluorotyrosines into the R2 subunit of *E. coli* Ribonucleotide reductase by expressed protein ligation. M.R. Seyedsayamdost, C.S. Yee, J. Stubbe, *Nat. Protoc.* 2007, **2**, 1225–1235.

Buffers. Assay buffer consists of 50 mM HEPES, 15 mM $MgSO_4$, 1 mM EDTA pH 7.6 at room temperature. For pH dependent studies, assay buffer consists of 50 mM MES (pH 5.2 – 6.6), 50 mM HEPES (pH 7.6 – 8.2), or 50 mM TAPS (pH 9.0), 15 mM $MgSO_4$, 1 mM EDTA.

Biochemical details

[Re_{356}]-β_2 construction. pET9d-His_6-C_{268}S-C_{305}S-nrdB encodes for the β subunit of RNR with modifications to include an *N*-terminal His_6 tag and serine substitution of two surface accessible cysteine mutations (C_{268}S and C_{305}S), and was used as a template for site-directed mutagenesis. The following forward and reverse primers were employed to introduce a Y_{356}C mutation into this construct.

F: 5' – AG GAA GTG GAA GTC AGT TCT T**G**T CTG GTC GGG CAG – 3'
R: 5' – CTG CCC GAC CAG A**C**A AGA ACT GAC TTC CAC TTC CT – 3'

Site-directed mutagenesis, amplification, and isolation of modified gene targets were performed as previously reported.[3] DNA sequencing was performed by the Dana-Farber/Harvard Cancer Center DNA Resource Core (Boston, MA), and the resulting chromatograms were analyzed in Geneious 6.1.3 (Biomatters, New Zealand).

RNR subunit expression, purification, reconstitution, and labeling. [Re_{356}]-β_2 was expressed, purified, reconstituted, and labeled as previously reported for [Re_{355}]-β_2.[3] Wild-type-β_2 (wt-β_2), wild-type-α_2 (wt-α_2), and Y_{731}F-α_2 subunits were prepared as previously reported.[2] Except for catalytic activity assays, experimental measurements were performed on HU treated β_2 subunits, in which the stable diferric-Y• cofactor is reduced and inactive (met-β_2). All α_2 proteins were pre-reduced with 30 mM DTT and treated with 20 mM HU to inactivate co-purifying wt-β_2. Protein purity was assessed by sodium dodecyl sulfate polyacrylamide gel electrophoresis (SDS-PAGE) with the resulting gels shown in Figure S2. Specific activity for each protein construct was measured by the standard spectrophotometric assay.[5,6]

2,3,5-F_3Y_{731}-α_2 expression and purification. 2,3,5-F_3Y_{731}-α_2 was prepared as previously reported with minor modification.[1] Specifically, following chromatography from the Ni-NTA column, the resulting eluent was diluted with lysis buffer (50 mM Tris, pH 7.6, 5% glycerol) to [$NaCl_{eluent}$] < 100 mM. The resulting solution was loaded onto a Q-Sepharose anion exchange column (CV = 25 mL) that had been pre-equilibrated with wash buffer (50

5 pH rate profiles of F_nY_{356}-R2s (n = 2, 3, 4) in *Esherichia coli* ribonucleotide reductase: Evidence that Y_{356} is a redox-active amino acid along the radical propagation pathway. M.R. Seyedsayamdost, C.S. Yee, S.Y. Reece, D.G. Nocera, J. Stubbe, *J. Am. Chem. Soc.* 2009, **128**, 1562–1568.

6 Pre-steady-state and steady-state kinetic analysis of *E. coli* class I ribonucleotide reductase. J. Ge, G. Yu, M.A. Ator, J. Stubbe, *Biochemistry* 2003, **42**, 10071–10083.

mM Tris, 100 mM NaCl, pH 7.6, 5% glycerol), washed with 4 CV of wash buffer, eluted with a shallow, linear gradient (100 to 400 mM NaCl, 40 CV), and collected in 1 min fractions (15 mL) under gravity flow. Fractions containing only full-length protein were pooled, concentrated, and pre-reduced in the same manner as wt- and $Y_{731}F$-α_2.

Biophysical measurements

The home-built nanosecond laser system is previously described,[7] and only modifications to the set-up are denoted below. Transient emission kinetics were measured on samples of 2.5 μM [Re_{356}]-β_2, 6.25 μM α_2 (as indicated), 1 mM CDP, 3 mM ATP in assay buffer, λ_{ex} = 355 nm, λ_{obs} = 600 nm. A laser power of 650 μJ/pulse (λ > 375 nm) was used as the pump signal to excite the sample (pre-equilibrated in a RT water bath for > 2 min). The resulting luminescence intensity was passed into the iHR320 spectrometer (Horiba), and averaged for display on the LeCroy oscilloscope. To ensure measurements were recorded from the linear response of the Hammamatsu photomultiplier tube (PMT, 1.00 kV), the slit width was adjusted so that the oscilloscope received a signal intensity < 2 mV. Slit widths ranging from 0.25 – 0.75 nm giving rise to signal intensities of 0.5 – 1.5 mV (as displayed by the oscilloscope) provided the most consistent lifetime measurements. The raw data for each independent sample was collected as two separate traces averaged over 500 laser shots each (2 × 500 sweeps), recruited into the kinetics program (developed by Bryce. A. Anderson), and saved as a data file (.dat). Results were plotted in OriginPro 8.5 SR1, and fit to a monoexponential decay function. Data and residuals are represented by circles as shown in Figure S4. The solid lines represent the monoexponential fit functions used in determining the measured mean lifetime (τ), and in calculating the uncertainty in and value of k_q as described in Eq. (S5).

To maintain consistency between independent sample preparations, reaction vessels were prepared from the addition of assay buffer that was 4× the desired final concentration of buffer components, followed by the addition of ATP and CDP, and α_2 variant. After supplementing the reaction tube with [Re_{356}]-β_2, 18 MΩ ddH2O was added to attain a final volume of 550 μL. After each laser trial (2 × 500 continuous laser shots), the flow cell cleaned with copious amounts of 18 MΩ cm−1 ddH2O, followed by reagent grade acetone. Low pressure N2 was used to dry the flow tubing and cuvette.

To minimize freeze thaw cycles between laser experiments, proteins were concentrated to achieve the highest soluble concentration (α_2: ~300 – 500 μM; β_2: ~1 mM), and stored in single use aliquots (~50 – 200 μL).

---

7 Deciphering radical transport in the large subunit of class I ribonucleotide reductase. P.G. Holder, A.A. Pizano, B.L. Anderson, J. Stubbe, D.G. Nocera, *J. Am. Chem. Soc.* 2012, **134**, 1172–1180.

Emission quenching data analysis

Individual traces resulting from the relaxation of [Re]* emission measured at 600 nm were fit to monoexponential decays. The assignment in the goodness of fit was evaluated by featureless residuals vs. time plots, reduced χ^2 value, and closeness to 1 adjusted R^2 value. Measurements were performed in triplicate under identical conditions between independent samples in order to obtain the standard deviation (σ) of the measured mean lifetime (τ). We estimate the uncertainty in τ for each $[Re_{356}]$-β_2:α_2 complex by evaluating for the standard error of the mean as defined by Eq. (S1),

$$\text{Standard Error of the Mean (SE)} = \frac{1.96\sigma}{\sqrt{n}} \tag{S1}$$

where n = the size of the sampling distribution. The SE (provided within parenthesis following an experimentally determined value) is reported to 95% confidence limits (2σ). The quenching rate constant, k_q, is calculated from the observed mean lifetime of [Re]* when $[Re_{356}]$-β_2 forms a complex with $Y_{731}F$-α_2 (τ_0) and either wt-α_2 or 2,3,5-F_3Y_{731}-α_2 (τ, as indicated) from Eq. (S2):

$$k_q = \frac{1}{\tau} - \frac{1}{\tau_0} \tag{S2}$$

The error in this function can be described in terms of the magnitude error in an inverse function as shown in Eq. (S3),

$$f(x) = \frac{1}{x} \tag{S3}$$

which is given by the magnitude of the derivative of the function given by Eq. (S4),

$$\Delta f(x) = \frac{1}{x^2}\Delta x \tag{S4}$$

where Δx is the standard error (Eq. S1) in the measurement of x. In the case of the measured mean lifetimes, $\Delta\tau$ corresponds to the standard error in τ. This error is propagated into the determination of Δk_q which represents the 2σ confidence interval for the calculated value of the quenching rate constant. The uncertainty in k_q is calculated according to Eq. (S5).

$$\Delta k_q = \sqrt{\left(\frac{1}{\tau^2}\Delta\tau\right)^2 + \left(\frac{1}{\tau_0^2}\Delta\tau_0\right)^2} \tag{S5}$$

(a)

(b)

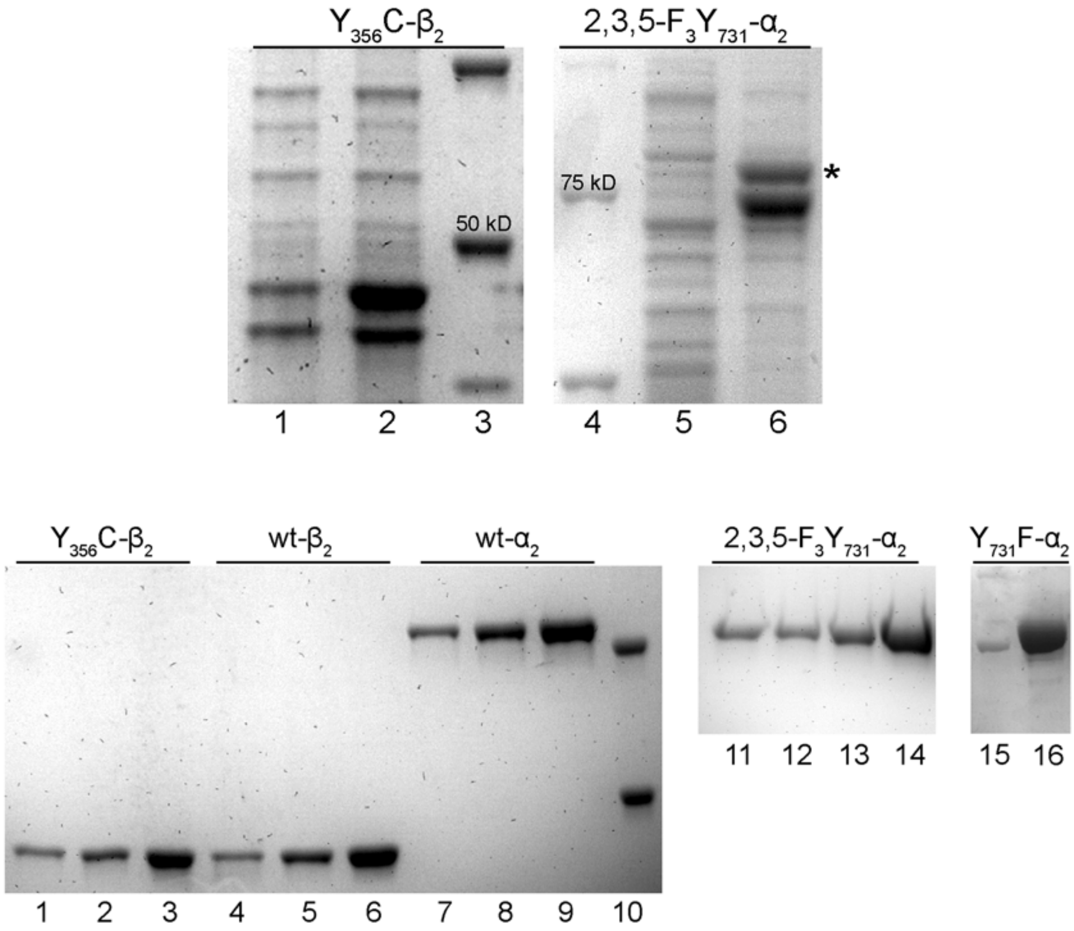

Figure S1. Expression of recombinant RNR subunits and assessment of protein purity by SDS-PAGE. (a) Expression gels of $C_{268}S$-$C_{305}S$-$Y_{356}C$-β_2 and 2,3,5-F_3Y_{731}-α_2. 1 mL aliquots of the pre-induction (OD_{600nm} = ~0.67) and post-induction cultures (OD_{600nm} > 3) were centrifuged for 3 min at 3,000 × g. The supernatant was decanted, and the resulting pellet was re-dissolved in a volume of ddH_2O that corresponded to the OD at 600 nm divided by a factor of 20. Lanes in $Y_{356}C$-β_2: **1** pre-induction; **2** post-induction; and **3** protein standard. Lanes in 2,3,5-F_3Y_{731}-α_2: **4** protein standard; **5** pre-induction; and **6** post-induction. The asterisk denotes the full-length band, which illustrates the suppression yield of 40% (supplemented with 0.7 mM 2,3,5-F_3Y). (b) Purity gels of RNR subunits. Dilute volumes of concentrated RNR protein were mixed with 2× Laemelli's buffer, boiled for 5 min, and loaded on a 7.5% Mini-PROTEAN TGX precast gel. With the exception of the $Y_{731}F$-α_2 variant (lane **16**, ~5 μg), all purified protein samples (as indicated above the wells) were loaded in succession of increasing amounts (0.5, 1.0, and 2 μg). At least one lane in each gel was dedicated to a protein standard (lane **10**, lane **15**), or authentic protein (lane **11**, His_6-wt-α_2). A constant electric current (180 V) was applied to the gel electrophoresis apparatus for ~45 min. Following removal from the pre-cast well plates, the gels were soaked in Coomassie Brilliant Blue, and destained overnight.

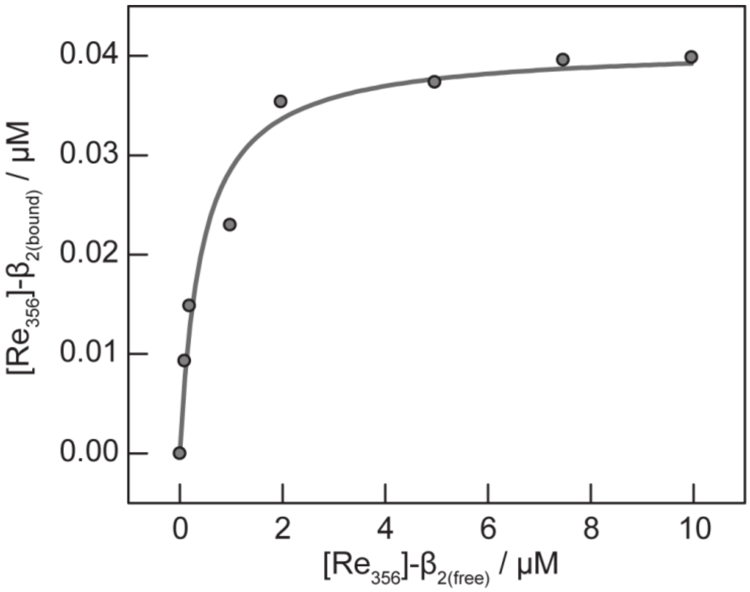

Figure S2. Competitive inhibition assay to determine K_D for $[Re_{356}]-\beta_2$ and wt-α_2. $[Re_{356}]-\beta_2$ was titrated against a solution of wt-RNR, substrate (CDP) and effector (ATP). Specific activity was measured using a spectrophotometric assay of NADPH consumption. Each 150 μL reaction contained: 0.2 μM wt-β_2, 0.1 μM wt-α2, $[Re_{356}]-\beta_2$ (0-10 μM), 30 μM TR, 0.5 μM TRR, 1 mM CDP, 3 mM ATP, 0.2 mM NADPH in assay buffer (pH 7.6). Data were fit to a hyperbolic equation; at these concentrations, $[\alpha_2:\beta_2]_{max}$ is 0.05 μM, using a K_D = 0.15 μM.[8]

[8] Proton–Coupled Electron Transfer in the Escherichia coli Ribonucleotide Reductase. M.C.Y. Chang, Ph.D. Thesis, Massachusetts Institute of Technology, 2004.

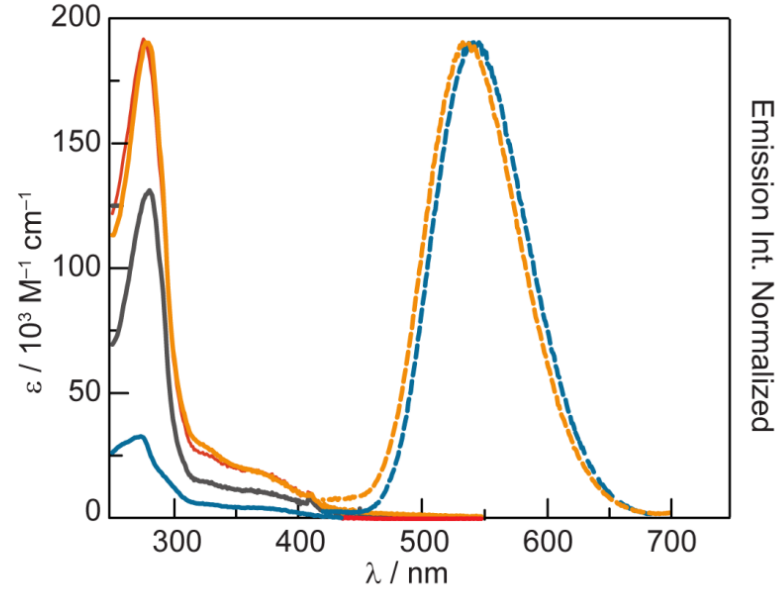

Figure S3. Spectroscopic comparison of [Re_{356}]-β_2, [Re]-Br, and reconstituted-$Y_{356}C$-β_2. Absorption (solid lines) and emission (dashed lines) spectra for [Re]-Br (blue); [Re_{356}]-β_2 (yellow); reconstituted $Y_{356}C$-β_2 (gray); and the simulated absorption spectrum of 2 [Re] molecules per β_2 (red). For RNR subunits, a concentration of 10 μM in assay buffer was used. Spectra for [Re]-Br were obtained from 50 μM in MeCN.

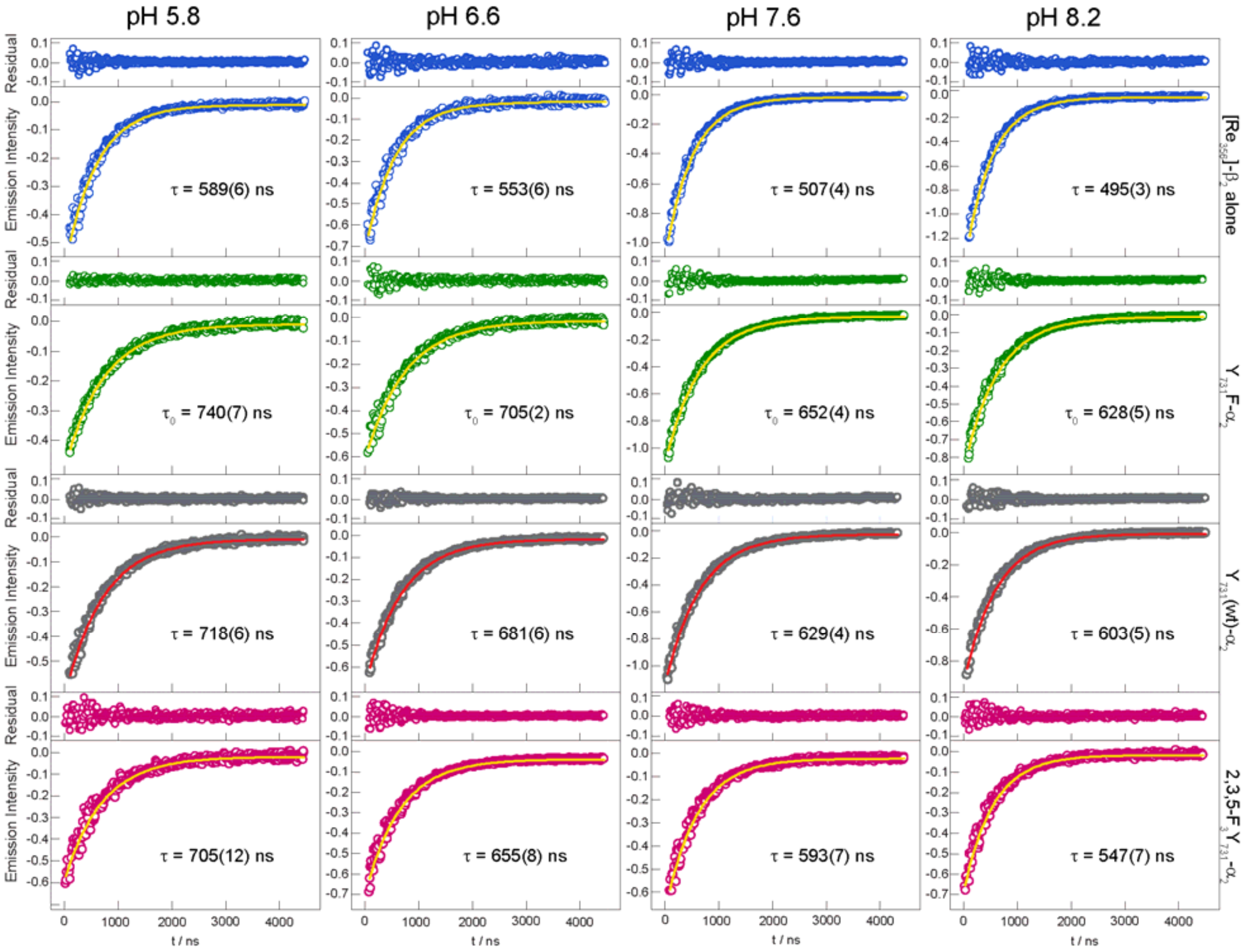

Figure S4. Representative transient decay traces and residuals of [Re_{356}]-β_2 alone (blue circles, ○), and in complex with $Y_{731}F$-α_2 (green circles, ○), wt-α_2 (gray circles, ○), or 2,3,5-F_3Y_{731}-α_2 (pink circles, ○). Each row represents a unique [Re_{356}]-β_2 interface, and each column represents the experimental pH. Lifetimes (τ, as shown) were determined from the monoexponential fit function as represented by solid lines. Residuals are provided directly above the corresponding decay trace.

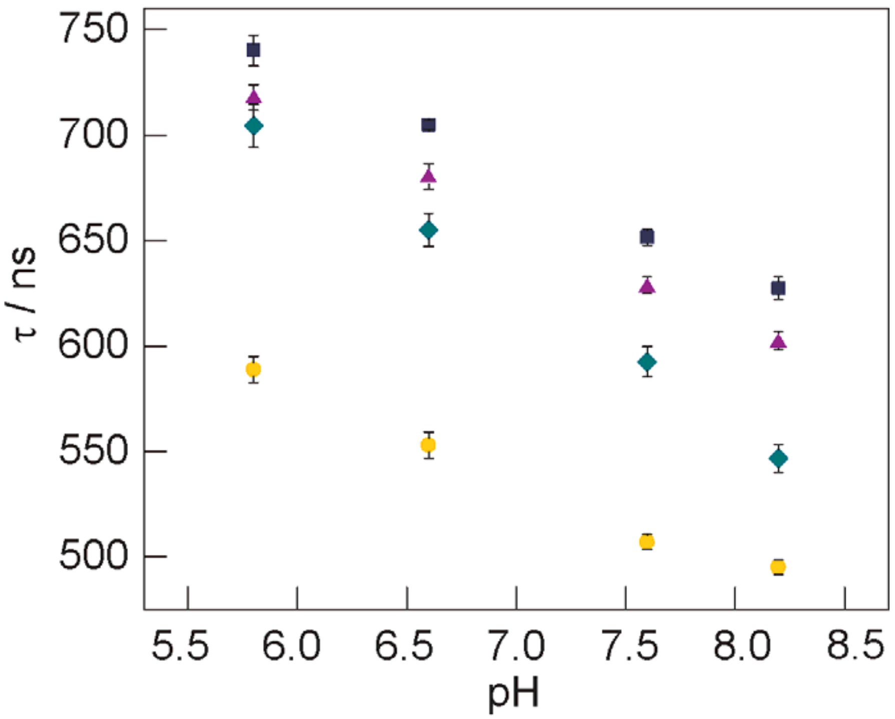

Figure S5. $[Re]^*$ lifetime of emission decay vs. pH of $[Re]^*$ for $[Re_{356}]$-β_2 alone (orange circles, ●) and in the presence of indicated α_2 variants: $Y_{731}F$-α_2 (dark blue squares, ■), Y_{731}(wt)-α_2 (purple triangles, ▲), or 2,3,5-F_3Y_{731}-α_2 (turquoise diamonds, ♦). Transient emission kinetics were measured on 2.5 μM $[Re_{356}]$-β_2, 6.25 μM α_2, 1 mM CDP, 3 mM ATP in assay buffer, λ_{exc} = 355 nm, λ_{obs} = 600 nm. The [Re]* decay in the absence of complexation to α_2 (orange circles, ●) decreases semi-uniformly as pH increases, indicating a slight dependence of [Re]* relaxation kinetics on pH within the photoβ_2 construct. A similar trend for the control complexes, $[Re_{356}]$-β_2:$Y_{731}F$-α_2 (dark blue squares, ■) and wild-type, $[Re_{356}]$-β_2:Y_{731}-α_2 (purple triangles, ▲) is observed, whereas the pH dependent change in lifetime for the $[Re_{356}]$-β_2:2,3,5-Y_{731}-α_2 complex (turquoise diamonds, ♦) is much steeper.

Table S1. Quenching rate constants corresponding to Y_{731} oxidation with [Re] at position 355 in the β_2 subunit calculated from lifetimes as reported in ref. 9.

β_{356}[a]	α_{731}[a]	$\tau_{600\,nm}$ / ns[b]	k_q / 10^4 s^{-1}	Enhancement
Y	Y	543(13)	14.7(6.6)	2.1(1.2)
Y	F	590(17)		
F	Y	696(5)	7.1(2.3)	
F	F	732(11)		

[a] Protein concentrations used for [Re_{355}]-β_2 and α_2 were 10 and 25 uM, respectively. [b] Laser power used was 2 mJ/pulse.

[9] Modulation of Y_{356} photooxidation in *E. coli* class Ia ribonucleotide reductase by Y_{731} across the α_2:β_2 interface. A.A. Pizano, L. Olshansky, P.G. Holder, J. Stubbe, D.G. Nocera, *J. Am. Chem. Soc.* 2013, **135**, 13250–13253.
